# A Novel Memory and Time-Efficient ALPR System Based on YOLOv5

**DOI:** 10.3390/s22145283

**Published:** 2022-07-14

**Authors:** Piyush Batra, Imran Hussain, Mohd Abdul Ahad, Gabriella Casalino, Mohammad Afshar Alam, Aqeel Khalique, Syed Imtiyaz Hassan

**Affiliations:** 1 Department of Computer Science and Engineering, Jamia Hamdard, New Delhi 110062, India; piyushbatra1999@gmail.com (P.B.); aahad@jamiahamdard.ac.in (M.A.A.); aalam@jamiahamdard.ac.in (M.A.A.); aqeelkhalique@jamiahamdard.ac.in (A.K.); 2 Department of Computer Science, University of Bari, 70125 Bari, Italy; gabriella.casalino@uniba.it; 3 Department of CS and IT, Maulana Azad National Urdu University, Hyderabad 500032, India; s.imtiyaz@gmail.com

**Keywords:** ALPR, YOLOv5, IoT, OCR, vehicle license plate detection and recognition, urban mobility

## Abstract

With the rapid development of deep learning techniques, new innovative license plate recognition systems have gained considerable attention from researchers all over the world. These systems have numerous applications, such as law enforcement, parking lot management, toll terminals, traffic regulation, etc. At present, most of these systems rely heavily on high-end computing resources. This paper proposes a novel memory and time-efficient automatic license plate recognition (ALPR) system developed using YOLOv5. This approach is ideal for IoT devices that usually have less memory and processing power. Our approach incorporates two stages, i.e., using a custom transfer learned model for license plate detection and an LSTM-based OCR engine for recognition. The dataset that we used for this research was our dataset consisting of images from the Google open images dataset and the Indian License plate dataset. Along with training YOLOv5 models, we also trained YOLOv4 models on the same dataset to illustrate the size and performance-wise comparison. Our proposed ALPR system results in a 14 megabytes model with a mean average precision of 87.2% and 4.8 ms testing time on still images using Nvidia T4 GPU. The complete system with detection and recognition on the other hand takes about 85 milliseconds.

## 1. Introduction

Traffic security and congestion represent some of the major problems in upcoming smart cities. Any contribution to help manage these cities will be beneficial for everyone. With the further development of smart cities and consequently intelligent transportation systems, efficient automatic license plate recognition systems are now required more than ever. These systems have gained much attraction due to their application in intelligent surveillance systems which have various use cases such as automated parking lot management, traffic surveillance, vehicular access control, etc., which represent emerging research areas under the scope of urban mobility. 

Internet of Things (IoT) devices are building blocks for smart cities. These are minute digital devices used in large quantities to complete data processing tasks. Although IoT processing devices such as Google Coral, Raspberry Pi, NVIDIA Jetson Nano, etc., are more powerful than typical IoT devices, they still lack continuous processing and cooling capabilities unlike traditional machine learning workstations. This paper aims to present the developed size and time-efficient system which can be deployed on small IoT processing devices, which can contribute to the development of smart cities.

Automated License Plate Recognition (ALPR) systems usually have two parts: license plate localization and character recognition. In the localization stage, the number plate is detected and cropped from the frame for character recognition. In the next stage, the cropped license plates are processed and given as input to a character recognition engine. Although there are plenty of ALPR systems in development, most of them are heavily dependent on high compute resources. In this paper, we present a system that is significantly smaller in size and can work on devices with low computing power. 

Deep neural nets have proven to be the best choice for object detection tasks. To build a robust deep learning model, a huge amount of labelled data are often required. However, collecting and labelling data of such magnitude is time-consuming and cost-ineffective. To overcome this issue, we adopted a concept called transfer learning [1]. In this technique, weights obtained from the pre-trained model are used to initialize a new custom detector. In our case, we used weights from the model trained on the Microsoft COCO [2], which is a dataset consisting of 91 classes with over 2.5 million labelled instances in 3.25 lakh images. This technique helped us to build a better-generalized model with a low training time.

Our approach consists of two subsystems. The first sub-system of our proposed system, which is used for license plate detection, is based on the recently released YOLOv5 [3] which is a time-efficient successor of You Look Only Once [4] advanced deep learning object detection architecture. For this sub-system, we chose the lightweight version of Yolov5, named v5small, which consists of 283 layers, 16.4 GFLOPS, and about 7 million parameters. For character recognition, we used an open-source OCR engine known as EasyOCR. It is an optical character recognition module that supports more than 80 languages, including Hindi, English, Chinese, Arabic, Dutch, and more. Moreover, this paper focuses more on license plate detection as EasyOCR itself is a state-of-the-art optical character recognition framework.

In this paper, the conceptual background regarding the technologies is given in Section 2. Section 3 discusses the previous work [5,6,7,8] completed in this field. Section 4 provides the methodology and implementation detail regarding the proposed model. Section 5 discusses the results obtained after the implementation. Finally, Section 6 discusses the conclusion and future work related to the research. 

## 2. Conceptual Background

This section presents a brief background on the frameworks and models used. It describes the different versions of YOLO and offers a brief introduction to the EasyOCR library.

### 2.1. YOLOv5

“You look only once” is an advanced algorithm for real-time object detection. It is the go-to architecture for many AI computer vision engineers. YOLOv1 [4] was released in 2016 and it was a leap forward in real-time object detection research. After 1 year, in 2017, YOLO9000 was released. It is commonly known as YOLOv2. This version was faster, better, and developed to be on par with the performance of Faster-R-CNNs. After that, another darknet-based version named YOLOv3 [9] was released. This iteration is the most popular and brought down the detection errors drastically. In 2020, researchers published a much-advanced version of Yolo called YOLOv4 [10]. This version incorporated new features such as Mosaic data augmentation, Cross mini-Batch Normalization (CmBN), Cross-Stage-Partial-connections (CSP), Weighted-Residual-Connections (WRC), Self-adversarial-training (SAT), and Mish-activation. Using all these new features, YOLOV4 achieved a whopping 65.7% AP50 on the Microsoft COCO [2] dataset. Within a few days of the release of YOLOv4, PyTorch-based YOLOv5 [3] surfaced.

**Figure 1:**
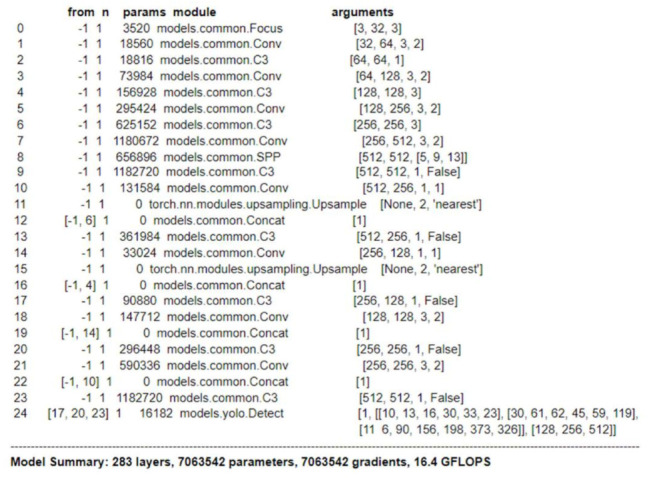
Architecture of YOLOv5s.

YOLOv5 is the newly released version of the Yolo family. It is a leading-edge deep learning algorithm that is very time performant and produces object detection models which are relatively smaller in size. For this paper, we chose its lightweight version called YOLOv5s (small). It is a 283-layers deep neural network with over 7 million trainable parameters. For more information about the layers, please refer to Figure 1 given below.

YOLOV5 is a single-stage object detector with three important parts, namely, the model backbone, neck, and head. In the first part, the model backbone, important features from the provided input image are extracted. In this process, cross-stage partial networks are used to identify information-rich features from the image. The neck helps the model to perform well on unseen data. It generates feature pyramids which help the model to generalize well. It also helps to detect objects of varying sizes and scales. Lastly, the model head is used to perform the end decision. It carries the features generated by the model neck and generates the output vectors with scores and coordinates for bounding boxes.

Moreover, YOLOv5 is significantly smaller in size than any of its predecessors, yet it performs on par with all of them, in some cases better, and it is user-friendly to train and deploy. All these characteristics make it the perfect candidate for our study. Further in this paper, we will demonstrate the performance metrics that we used and the results obtained by our model.

### 2.2. EasyOCR

**Figure 2:**
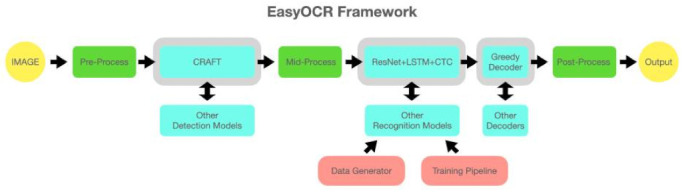
EasyOCR pipeline [12].

EasyOCR is a state-of-the-art OCR library that is efficient and supports over 80 languages. It is built using python and Facebook’s PyTorch framework. It carries out detection using a character region awareness algorithm commonly known as CRAFT [11] which is a neural network-based scene text detection model. For recognition, EasyOCR uses a CRNN which consists of ResNet for feature extraction, LSTM sequence labelling, and CTC decoding. Figure 2 represents the exact working pipeline of EasyOCR. This pipeline also includes some pre-processing steps which make this engine one of the best [12].

Although this OCR engine can detect and recognize more than 80 languages, we used it in the English configuration. After detecting the license plate in the input image, we cropped and provided as the input to this OCR engine to obtain the license plate number.

## 3. Literature Review

Vehicle license plate detection and recognition have been compelling areas of research for a long time. Computer vision researchers have devised many different approaches to solve the task at hand. 

Over the past few years, many researchers have addressed the license plate detection task. For example, the authors of [13] used ResNet-50-based Faster-RCNN for Indian license plate detection and achieved a very high mAP. Mean Average Precision (mAP) is a commonly used measurement of precision for object detection models and measures the crossing of the predicted bounding box with the labelled bounding box. 

The authors of [14] proposed a Fast-YOLO [15] and YOLOv2 [16]-based detector. In this approach, the authors trained two CNNs, first for vehicle detection and another for license plate detection. Their results suggested that Fast-Yolo had impressive results in both tasks. On the other hand, the authors of [17] used an exemplar SVM-based approach along with Fast and Faster-RCNN, depicting that RCNN was better suited for real-time detection. The authors of [18] also presented a YOLOv2 and Fast YOLO-based single cascaded CNN to detect both car frontal car views and licence plates, achieving high recall and precision rates. The authors of [19] used 500 images to train an AlexNet neural network and to run it on the Jetson TX1 board. Although they achieved a high accuracy, their testing set only consisted of 64 images. The authors of [20] proposed a combined deep learning end-to-end framework that eliminates the need for training separate systems for detection and recognition tasks. The authors of [21] presented a two-step approach based on InceptionV2 FasterRCNN and Long Short Term Memory (LSTM) Tesseract and managed to achieve a high accuracy in the detection task [22] using OpenALPR, which is a C++ based commercial license plate recognition library, to manage parking lot access using Raspberry Pi. The author of [23] presented a modified sliding window-based SWSCD-YOLO system that was tested for different climate conditions. This system attained test speeds of 800 ms to 1 s. 

The authors of [24] used DNN to recognize the license plate numbers of cars. They used a single pass to achieve the detection and recognition of the same. In [25], the authors proposed a three-step approach for the detection and recognition of license plate numbers. They used YOLOv3. Their approach was devised to recognize number plates for different countries. The authors of [5] reviewed the state-of-the-art ALPR systems. They used different characteristics for comparison including datasets used, colors of plates, number fonts, etc. In [6], the authors proposed a CNN-based ALPR system. The claim was to achieve 98% recognition precision. The authors in [7] proposed an optimal K-means method using CNN for the automatic recognition of number plates. They performed a simulation to prove the effectiveness of their approach. 

In [8], the authors proposed a statistical PRS. They used three cascading modules for the recognition and identification of number plates. The authors of [26] proposed a new method for detecting number plates using adaptive image segmentation and a probabilistic neural network (PNN) mechanism. They claimed to have achieved about 86% accuracy in prediction. The authors of [27] proposed a CNN-based approach using MD-YOLO for multi-direction car LPR. An empirical evaluation was conducted to prove the improvement achieved in their approach. In [28], the authors proposed a method to recognize the car make and model through traffic cameras. The authors of [29] proposed a method using vertical edge detection for identifying car license plates. In [30], the researchers proposed an approach to identify license number plates for Italian vehicles. The authors claimed to have received a recognition accuracy of around 91%. An approach for real-time detection and segmentation of license plates was proposed in [31]. They claimed to have achieved an accuracy of 95% for classifying the digits. There are other researchers [32,33,34,35,36,37,38,39,40,41] who have proposed different approaches for the detection of license plates of vehicles. Table 1 provides a summary of this research.

All these results further encouraged us to use a novel YOLOv5-based deep learning approach for license plate detection in our system. Although YOLOv5 is fairly new, its performance in license plate detection tasks is remarkable.

## 4. Materials and Methods

This section presents a description of the datasets used, the performance metrics, and the methodology adopted for performing the task of automatic license plate recognition. A brief description of the training, testing, and validation data has been provided. The performance metrics such as mean average precision have been described in detail.

### 4.1. Dataset

**Figure 3:**
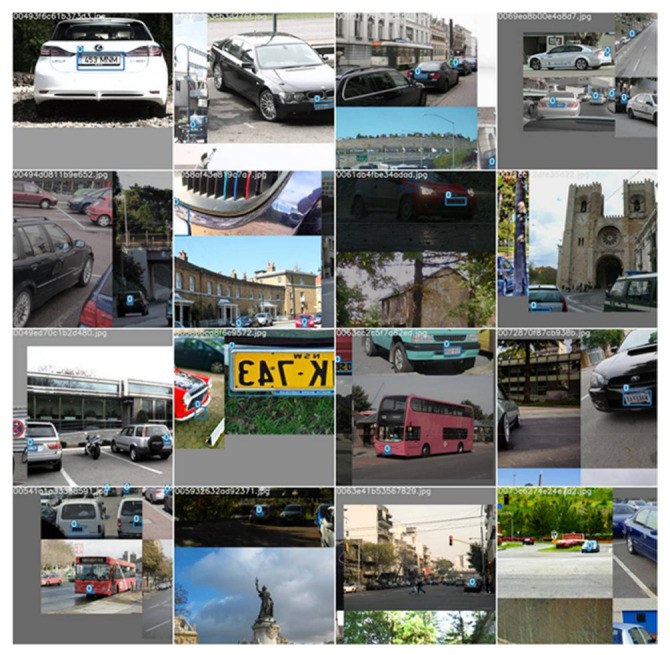
Random samples from the dataset (https://www.kaggle.com/datasets/dataturks/vehicle-number-plate-detection, accessed on 5 June 2022) (https://storage.googleapis.com/openimages/web/index.html, accessed on 5 June 2022).

The dataset that we used for this paper is a combination of license plate data from the Google open Images dataset and the Indian number plates dataset that is available on Kaggle. We put all the images from both datasets in a single folder and randomly split them into training, validation, and testing sets. Our dataset is available on Kaggle for the research community to use for further enhancement and sound comparison. This dataset contains 5991 images of vehicles in jpg format. Figure 3 represents some random samples from the aforementioned dataset.

The dataset was divided into 3 folders, namely train, test, and validation. The training set contained 4201 samples which roughly constituted 70% of the dataset. The validation set that was used to validate the model performance while training contained 1188 images which composed nearly 20% of the database. Lastly, the test dataset which was used to perform the final testing of the model contained 602 images.

All the images in this dataset have a text label file embedded with them. This label file contains bounding box coordinates for a number plate in YOLO annotation format. YOLO accepts the labels in a certain format given below:

<object-id> <center_x> <center_y> <width> <height>(1)

where:

object-id: number corresponding to object category;
Center_x and ceter_y: normalized center point of the bounding box;
Width and height: normalized width and height of the bounding box.

To create a robust system, our dataset contained images from different angles and different climate conditions. All these images were of different sizes and resolutions but the final input image to the model was provided in 640 pixels.

### 4.2. Performance Metrics

For object detection tasks, mAP or mean average precision is often the performance metric of choice for many computer vision practitioners. Object detection tasks include identifying the relevant object in the images and then stratifying it into known classes. These tasks make predictions in the form of object class labels and a bounding box surrounding the object itself. Precision and recall are the two-core metrics that together were used to evaluate the mAP or performance of the model.

Precision is a measure of quality. It describes how often our model guessed correctly from all the guesses. Perfect precision means every guess is correct. On the other hand, recall is a measure of quantity; it indicates whether our model predicted the same result as expected or not. Perfect recall indicates that a model has made the correct number of predictions or has given full coverage.

Precision = TP/(TP + FP)(2)

Recall = TP/(TP + FN)(3)

where:

TP: True positives;
TN: True negatives;
FP: False positives;
FN: False negatives.

The mAP was calculated by computing a series of precision-recall curves (AP) over varying Intersection over Union (IoU) levels. IoU is another performance metric that measures the accuracy of the bounding box on a particular task. In other words, IoU measures the overlap between 2 annotations. First, the average precision per class is calculated, and then it is averaged over all object categories to obtain mAP.

In this study, we evaluated mAP@0.5 and mAP@0.5:0.95 which means mean average precision with an IoU threshold of 0.5 and mean average mAP over multiple IoU thresholds, from 0.5 to 0.95, respectively.

### 4.3. Methodology

**Figure 4:**
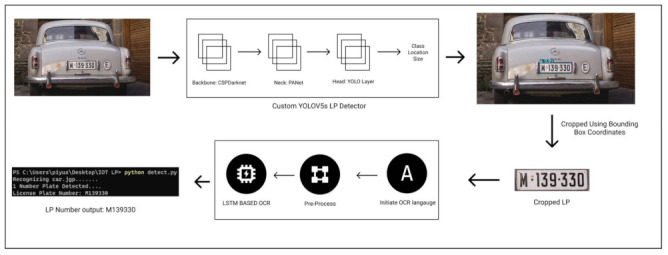
Overview of the methodology.

This proposed ALPR framework consists of 2 subsystems: license plate detection and character recognition (Figure 4). For the first sub-system, the small and lightweight version of YOLOv5 is used. Encouraged by the success of previous YOLO models, in this paper, we used the YOLOv5s model for license plate detection. For training this model we used transfer learning and initialized the model with weights learned from the Microsoft COCO [2] dataset. This dataset contains 91 classes of objects with over 2.5 million labelled instances in 3.25 lakh images. In this approach, we adapted the features learned from a more extensive dataset and employed them for our use. Utilizing such learned features allowed us to build a robust and high-performing model from the available data. Along with this, we used an SGD optimizer and trained our model for 100 epochs. 

This license plate detection subsystem takes input in the form of still images or videos or a network stream and detects the license plate in every frame. The output of this system is the coordinates for the detected bounding box. After detecting the license plate from the image, we used OpenCV [46] to further process it. Using the bounding box coordinates from the first module, we cropped out the license plate and fed it to the next subsystem. This subsystem utilizes the power of the LSTM-based EasyOCR engine to recognize the license plate number.

The aforementioned system takes about 4.8 ms for license plate detention and about 80 ms for detection on NVIDIA T4 GPU. Due to this fast inference time, it is an ideal system for IoT processing devices such as Jetson Nano or Google coral, or Intel NCS.

## 5. Results and Discussion

From Table 2 we can see that our model achieved an mAP of 88.4% on our validation set and 87.2% on our test set. This mAP was calculated over the intersection threshold (IoU) of 0.5. Our model also achieved a very high recall and precision. Along with achieving an mAP, our resultant model was only 14 megabytes with a testing time of 4.8 ms. This inference time was considerably fast and could help to develop a robust real-time ALPR system.

where:

R = Recall
P = Precision
mAP = Mean Average Precision with an IoU threshold of 0.5
mAP.95 = mean average mAP over multiple IoU thresholds, from 0.5 to 0.95; mAP@[0.5:0.95]

Table 3 shows the mAP values obtained using the model YOLOv5s. As we intended to deploy this proposed model on an IoT processing device, we also compared our model to the tiny version of darknet-based YOLOv4. We trained a tiny YOLOv4 model on the same dataset to illustrate a sound comparison. As you can see from Table 4, Tiny YOLOv4 achieved an mAP of 82.68% when trained and tested on the same dataset. It also produced a 22 MB model. Moreover, the testing time on the tiny yolov4 model was dramatically higher, 23.1 ms, than our proposed model.

We compared our results with other work that has been completed using similar architecture but different datasets. Due to the novelty of YOLOv5, not a lot of research has been undertaken using it. Table 4 and Table 5 shows the comparison between the work in [42,47] and our model. For a sound comparison, we used their trained model and tested it on our dataset.

These results show that our approach outperforms the previous work. The reason for this drastic performance difference might be that the dataset used by the author of [47] consisted of about 300 images. Some systems use OpenCV contours to detect the license plate [48], while others implement deep learning neural nets for plate localization.

Our proposed methodology for license plate detention achieved promising results. With some further changes, this system could be deployed for real-time detection.

## 6. Conclusions

This study aimed to develop a framework for license plate detection and recognition. Our model was intended for IoT processing devices. We applied the concepts of transfer learning and trained a small YOLOv5 model. We presented our results in terms of license plate detection as we used a state-of-the OCR recognition engine to carry out the character recognition task. Our results suggest that YOLOv5s is a very capable architecture and produces a time and memory-efficient model. Our approach resulted in a 14 megabytes model, which is appropriate for IoT devices with less memory. When tested, it performed well with a mean average precision of 87.2% and 4.8 ms testing time on still images using Nvidia T4 GPU.

Our model achieved a high mAP. Among other advantages, we achieved a low response time with a higher accuracy. However, there is still room for improvement. The two major limitations of our system are its ability to work under low visibility and night conditions and the lack of real-world performance measurements. In future work, we plan to modify the underlying architecture of this license plate recognition system and mold it in a way highly suitable for low processing devices. We also intend to train and test our model for low visibility conditions so that it works seamlessly during the night and bad weather. Additionally, we would also train a deep neural net for character recognition; hence, eliminating the need to use an external OCR engine. A lightweight OCR system would help us present a single end-to-end ALPR solution for IoT devices. Lastly, we intend to deploy this model on an IoT processing device and check its real-time performance.

## Figures and Tables

**Table 1:** Summary of the state-of-the-art research in ALPR [24,25,26,27,28,29,30,31,32,33,34,35,36,37,38,39,40,41,42,43,44,45].

Sr No	Ref	Technique Used	Dataset Used	Accuracy Achieved
1	[24]	Unified DNN	CarFlag-Large, China	97.13%
2	[25]	YOLOv3 and YOLOv3-SPP	KarPlate, Korea	98.93%
3	[6]	Multi-task Convolutional Neural Networks (MTCNN)	Chinese City Parking Dataset (CCPD)	98%
4	[7]	OKM-CNN (Deep Learning-based K-Means) CNN	Stanford Cars dataset, FZU Cars Dataset, HumAIn 2019 Dataset	98.1%
5	[8]	Cascade Framework	SeeCar library	98.25%
6	[26]	two-layer probabilistic neural network (PNN)	Self- created sample set	86%
7	[27]	CNN-based MD-YOLO	ImageNet dataset and Application Oriented License Plate (AOLP) dataset	F Score 99.5%
8	[28]	Image processing part-based detector mechanism	Self-created dataset	99.4%
9	[29]	Vertical Edge Detection	Self-created dataset	91.6%
10	[30]	Image processing	Self Created Dataset	91%
11	[32]	Two-Step Key frames identification approach	Video Dataset	F score 91%
12	[33]	(YOLO)-darknet deep learning framework using sliding-window approach	AOLP dataset	78%
13	[34]	Two-stage Convolutional Neural Networks (CNNs) using YOLOv3 framework	JALPR dataset	87%
14	[35]	Deep Learning based real-time video monitoring	UFPRALPR dataset, SSIG-SegPlate dataset, and Low-Quality Plate-Videos dataset	95.83% for the AOLP dataset and 98.9% for CCPD datasets
15	[38]	mask region convolutional neural networks	AOLP, Caltech dataset	99.3% on AOLP and 98.9% on Caltech dataset.

**Table 2:** Dataset Details.

	Train	Validation	Test	Total
License Plate	4201	1188	602	5991
	70.12%	19.83%	10.05%	

**Table 3:** Results.

Model	Epoch	Test (%)				Validation (%)				Size	Avg Test Time
		mAP	mAP.95	R	P	mAP	mAP.95	R	P		
YOLOv5s	100	87.2	46.5	82.2	88.2	88.4	49.5	84.3	87.8	14 MB	4.8 ms

**Table 4:** Comparison between YOLOv5s and tiny YOLOv4.

Model	Test mAP	Validation mAP	Size	Test Time
YOLOv5s	87.2%	88.4	14 MB	4.8 ms
Tiny YOLOv4	82.68%	83.95	22 MB	23.1 ms

**Table 5:** Comparison with other work completed with YOLOv5.

Model	mAP@0.5	mAP@0.5:0.95	Recall	Precision
Our model	87.2%	46.5%	82.2%	88.2%
Other work [42]	9.1%	2.4%	18.3%	17.9%

## Data Availability

Not applicable.

## Abbreviations

**Abbreviation**	**Explanation**
ALPR	Automatic number-plate recognition
LSTM	Long Short-Term Memory
OCR	Optical Character Recognition
YOLO	You Only Look Once
IoT	Internet of Things
COCO	Common Objects in Context
CRAFT	Character-Region Awareness For Text detection
CRNN	Convolutional recurrent neural network
RCNN	Regions with convolutional neural networks
DNN	Deep neural networks
MTCNN	Multi-Task Cascaded Convolutional Neural Networks
SGD	Stochastic Gradient Descent
mAP	mean average precision
